# Supporting Neurodiverse Junior Doctors: Challenges, Strategies, and Policy Implications for Inclusive Medical Training

**DOI:** 10.7759/cureus.104746

**Published:** 2026-03-05

**Authors:** Mariasole Barel, Amir Javaid

**Affiliations:** 1 Psychiatry, Burjeel Medical City, Abu Dhabi, ARE

**Keywords:** adhd, autism, healthcare policies, junior doctors, medical education, neurodiversity, psychiatry

## Abstract

Neurodiversity encompasses natural variations in brain function, including autism spectrum disorder (ASD), attention-deficit/hyperactivity disorder (ADHD), dyslexia, and dyspraxia. These differences influence cognitive processing, communication, and sensory perception, shaping how individuals engage with demanding clinical environments. In medicine, junior doctors face intense workloads and high emotional demands, which may amplify difficulties associated with neurodivergent traits. This editorial highlights the challenges experienced by neurodiverse junior doctors and reflects on the need for inclusive clinical and educational practices. Drawing on professional guidance from bodies such as the General Medical Council and the National Institute for Health and Care Excellence (NICE), this article discusses systemic gaps in recognition, limited accommodations, and persistent stigma within medical training. It emphasizes practical strategies, including structured mentorship, predictable scheduling, assistive technologies, and organizational awareness programs, and underscores institutional responsibilities and policy implications. By adopting inclusive frameworks aligned with established professional standards, healthcare organizations can better support neurodiverse clinicians, improve retention, and enhance patient-care quality.

## Editorial

The medical profession remains one of the most cognitively and emotionally demanding career paths, requiring sustained attention to detail, complex clinical reasoning, rapid decision-making, and continuous emotional regulation. Junior doctors must navigate rotating schedules, hierarchical training systems, unpredictable clinical environments, and high-stakes interpersonal interactions while maintaining patient safety. For neurodiverse doctors, those with neurological differences such as autism spectrum disorder (ASD), attention-deficit/hyperactivity disorder (ADHD), dyslexia, or dyspraxia, these challenges may be amplified by variations in cognitive processing, executive functioning, sensory sensitivity, and social communication.

The concept of neurodiversity refers to the understanding that variations in neurological development, such as ASD, ADHD, dyslexia, and dyspraxia, represent natural forms of human diversity rather than pathological deficits. This framework reframes such conditions as differences in cognitive functioning that may confer both strengths and challenges [1]. Approximately 15-20% of the global population is estimated to be neurodivergent [2]. Despite this prevalence, medical culture has historically emphasized conformity, endurance, and uniform performance standards, leaving limited institutional space for cognitive variation. As a result, neurodivergent trainees may experience under-recognition, stigma, and preventable attrition within healthcare systems.

Embracing neurodiversity within medicine offers tangible benefits. Distinct cognitive styles contribute to enhanced pattern recognition, analytical precision, creativity, and empathy. This editorial examines the biological underpinnings of neurodevelopmental variation, the lived and structural barriers faced by neurodiverse junior doctors, and evidence-based institutional strategies that promote equitable participation in medical education and clinical practice.

Biological foundations of neurodevelopmental diversity

Neurodevelopmental differences arise from multifactorial origins involving genetic predisposition, environmental influences, and neurobiological processes [3]. Genetic variations affecting synaptic connectivity, cortical development, and neurotransmitter systems have been associated with ADHD and autism spectrum conditions. Perinatal complications, including hypoxic injury or birth trauma, may alter neural pathway maturation. Emerging research highlights the role of maternal immune activation and inflammatory pathways in shaping fetal neurodevelopment. Prenatal infections and early childhood illnesses may further influence neuronal maturation, while traumatic brain injury during critical developmental periods can modify cognitive networks. Nutritional factors, particularly deficiencies in folate, iron, and omega-3 fatty acids during early brain development, may also contribute to variation in attentional and cognitive regulation [3]. 

These mechanisms underscore that neurodiversity reflects biological heterogeneity rather than uniform pathology. Recognizing this heterogeneity is essential for designing inclusive educational and professional systems that accommodate cognitive diversity rather than suppress it.

Neurodiversity in medical practice

Neurodiversity emphasizes that differences in attention, communication, and sensory processing represent normal variation within human populations [1]. Within medicine, neurodivergent professionals frequently demonstrate distinctive strengths. Autistic clinicians may exhibit strong logical consistency, exceptional pattern recognition, and meticulous attention to detail. Individuals with ADHD often display creativity, adaptability, high energy, and the capacity for intense hyperfocus in stimulating environments. These traits may enhance diagnostic reasoning and innovative problem-solving [3].

However, rigid workplace structures can inadvertently disadvantage such cognitive styles. Unpredictable shift rotations, frequent task-switching, high sensory stimulation, and informal communication norms may create barriers unrelated to clinical competence. These structural mismatches highlight the need for organizational adaptation rather than individual remediation.

Structural and cultural barriers in clinical training

Despite their strengths, neurodivergent junior doctors frequently encounter disproportionate barriers during training [4]. Rotating schedules, multitasking demands, and administrative overload may overwhelm executive functioning in trainees with ADHD. Rapid verbal instructions during ward rounds may disadvantage individuals who process information sequentially or visually. Differences in eye contact, tone, or social nuance may be misinterpreted as disengagement or unprofessionalism. Sensory overload from noise, bright lighting, and chaotic clinical environments can contribute to fatigue and anxiety. Furthermore, fear of stigma or perceived career repercussions often discourages disclosure and accommodation requests.

Workforce data indicate that neurodivergent professionals remain underrepresented in leadership positions and experience elevated burnout rates [4]. The Royal College of Psychiatrists (RCPsych) Position Statement PS05/23 emphasizes that inclusive training practices benefit all doctors, not solely those who disclose neurodivergent conditions. Similarly, the National Institute for Health and Care Excellence (NICE) guideline NG142 underscores organizational responsibility for workplace mental well-being and early intervention [5]. These frameworks shift responsibility from individual resilience toward systemic inclusion.

ADHD in junior doctors

ADHD is characterized by patterns of inattention, impulsivity, and hyperactivity that may interfere with sustained organization and task management [3]. In the clinical setting, junior doctors with ADHD may struggle with documentation backlogs, frequent interruptions, and prolonged procedures requiring sustained concentration. Stress, fatigue, and sleep disruption can exacerbate symptoms.

Supportive strategies include structured task breakdown, predictable scheduling, visual organizational tools, and protected short breaks during extended shifts. Consistent, practical feedback delivered constructively rather than punitively enhances confidence and reduces performance anxiety. Pharmacological therapy and cognitive-behavioral strategies demonstrate clinical efficacy; however, workplace understanding remains a critical determinant of success [3].

Autism in junior doctors

Autism spectrum conditions involve differences in social communication, sensory processing, and cognitive style [3]. Autistic trainees may interpret language literally, find unwritten social norms confusing, and experience distress in unpredictable or overstimulating environments. Research indicates that autistic adults experience disproportionately high rates of underemployment, despite educational attainment. Within medicine, autistic doctors report elevated stress but also describe strengths including precision, ethical consistency, empathy, and analytical focus [5]. 

Institutional support measures include clearly written expectations, structured teaching interactions, sensory-calming spaces, and designated mentors trained in autism awareness. Predictability and consistency significantly reduce anxiety and improve performance. When supported appropriately, autistic clinicians demonstrate exceptional patient rapport and sustained focus, particularly in procedural and analytical specialties.

Institutional accommodation and inclusive practice

Policy bodies consistently advocate person-centered accommodations within healthcare systems. NICE guideline NG142 highlights the responsibility of organizations to foster psychological safety and early support [6]. The World Health Organization’s Equity and Inclusion Framework for Health Workforce Diversity emphasizes measurable inclusion outcomes and workforce-wide audits [7].

Effective institutional interventions include structured learning models that divide complex tasks into predictable steps, advance scheduling notifications to reduce uncertainty, assistive technologies such as digital task organizers and speech-to-text tools, and environmental modifications, including quiet documentation spaces and reduced sensory clutter. Department-wide education programs that normalize neurodiversity discussions reduce stigma and promote an inclusive culture.

Inclusive practice requires both procedural reform and cultural transformation. Senior clinicians play a decisive role in modeling openness and acceptance. Transparent communication and individualized learning plans foster higher confidence and lower burnout among neurodivergent trainees.

Future directions

Future research should quantify the impact of inclusive interventions on objective outcomes such as examination performance, retention rates, burnout indices, and patient satisfaction. Comparative analyses across specialties and geographic regions may reveal context-specific barriers and facilitators. Standardized institutional metrics for measuring inclusivity should be integrated into accreditation frameworks. Longitudinal cohort studies examining mentorship outcomes among neurodivergent clinicians would further clarify best practices. Interdisciplinary collaboration between psychiatry, occupational medicine, and medical education research can refine evidence-based training models.

Conclusions

Neurodiverse junior doctors represent an underutilized yet highly valuable component of the medical workforce. Their cognitive diversity enriches diagnostic reasoning, innovation, and empathy. However, without structural and cultural adaptation, healthcare systems risk perpetuating inequity and preventable burnout.

Inclusion is not solely an ethical obligation but an operational necessity. Implementing evidence-based accommodations, including structured mentorship, predictable scheduling, and assistive technologies, enhances retention, performance, and patient care. Alignment with national and international frameworks such as NICE NG142 and the WHO Equity and Inclusion Framework transforms neurodiversity awareness into institutional competency. Healthcare systems that embrace cognitive diversity cultivate safer, more reflective, and more resilient clinical environments.
